# German Soldiers' Needs to Clarify Open Aspects in Their Life, to Talk About Fears and Worries, and to Forgive and to Be Forgiven as a Matter of Life Reflection

**DOI:** 10.3389/fpsyt.2018.00582

**Published:** 2018-11-13

**Authors:** Arndt Büssing, Daniela Rodrigues Recchia, Loren L. Toussaint

**Affiliations:** ^1^Professorship Quality of Life, Spirituality and Coping, Faculty of Health, Witten/Herdecke University, Herdecke, Germany; ^2^Department of Psychology, Luther College, Decorah, IA, United States

**Keywords:** soldiers, forgiveness, moral injury, stress perception, PTSD symptoms, life satisfaction

## Abstract

**Background:** In missions, soldiers are confronted with difficult situations which may impair their physical and mental health. As the resulting problems are commonly regarded as stigmata, soldiers may obviate talking about their experiences and try to oppress them. It was aim of this study to clarify whether soldiers do perceive needs to reflect back on life, to seek release from “open aspects” of their life, and to talk with others about fears and worries, to forgive others or to be forgiven. Further we intended to clarify whether these needs were related to stress perception, post-traumatic stress disorders (PTSD) symptoms and reduced life satisfaction on the one hand, and religious trust as a resource to cope on the other hand.

**Methods:** Cross-sectional survey of 1,097 German soldiers (92% men). Existential/spiritual needs and mental health indicators, including stress perception, PTSD symptoms, life satisfaction, were assessed using standardized questionnaires.

**Results:** For 30% of soldiers it was important to reflect on life, 23% had a strong need to clarify open aspects of life, 30% had a strong need to talk with others about their fears and worries, 13% had strong needs to forgive, and 13% had a strong need to be forgiven. Soldiers' needs to clarify open (and probably conflicting) aspects of life were moderately related to their intention to forgive others and to be forgiven (*r*s > 0.35). Soldiers treated in the hospital for psycho-mental trauma had significantly higher needs scores than soldiers still serving on active duty, particularly for the need to talk with others (*F* = 39.1; *p* < 0.0001) and to be forgiven (*F* = 26.0; *p* < 0.0001). Across all soldiers the best predictors of these needs were PTSD symptoms and stress perception, albeit with relatively weak predictive power (βs < 0.25; *R*^2^s < 0.24).

**Conclusions:** The process of life reflection and subsequent intention to solve conflicting situations and experiences can be considered a process of coping with one's own failures, guilt, and mistakes. It should be noted that these needs were significantly stronger in soldiers with trauma. Addressing unmet needs may help them to communicate and to reject the stigma of “weakness.”

## Introduction

In missions, soldiers are confronted with difficult situations which may impair their own physical and mental health. For soldiers it was stated that “the danger of spiritual and moral trauma is real, and it can initiate a downward spiral of physical, psychological, and behavioral problems in the service member” (1). Soldiers with these types of problems are commonly stigmatized (2, 3) even though help seeking behavior is generally encouraged and chaplains and other mental health service providers are available to offer support (4). As a result of being stigmatized soldiers may obviate talking about their experiences and, moreover, try to oppress them. Such burdening experiences may persist and interfere with adaptive coping strategies for dealing with Post-traumatic Stress Disorders (PTSD), or develop into a “moral injury.” Moral injury results from “acts that transgress deeply held moral beliefs” (5) with resulting “feelings of shame, grief, meaninglessness, and remorse from having violated core moral beliefs” (6). In these cases soldiers may either decide to call for professional help (i.e., psychologists, chaplains) or they may try to manage the situation by themselves either by ignoring the problem, suppressing emotions, or attempting to solve the underlying problems. The numbers of soldiers receiving counseling or therapy for mental health issues or substance abuse therapy is estimated to be 17–21% (4, 7). Morgan et al. (4) found that the most often stated reasons for mental health consultations were problems with the family, depression and anxiety, stress and anger management.

When soldiers are actively aware of these problems and are able to talk with others about their perceptions, it is much easier to provide support and help. However, when the perceptions are emotionally “separated” and not emotionally “processed,” it is much more likely that they do not talk about their problems to avoid the stigma of “weakness.”

It was thus recently suggested that soldiers' psychosocial, existential and spiritual *needs* should be addressed, instead of assessing and treating only their mental health conditions (i.e., depression, anxiety, PTSD symptoms) (8). Research with the *Spiritual Needs Questionnaire* (SpNQ) in a sample of German soldiers has shown high levels of psychosocial, existential and spiritual needs and found that particularly the needs to be connected with partner and family and to find “inner peace” were of relevance and less so religious or existential needs (9). An important theme in that study was soldiers' needs to communicate their own fears and worries (9), which may be a helpful means to find states of inner peace.

It is worthwhile to focus on soldiers' needs (either actively expressed or not) to reflect back on life and clarify open aspects of their lives and to talk with others about fears and worries. These fears and worries may be due to either disturbing experiences during their missions or interpersonal conflicts with partners, comrades, or superiors. When soldiers experience guilt, failure, shame and ultimately moral injury, they may have forgiveness needs. When they experience other persons' failures or interpersonal conflicts, they may need to forgive and thus to resolve the conflicts by starting a process of reframing perceived harm and reducing resentment.

Interpersonal conflicts and violence may have long-term effects in the life of the offended or injured person. Meanwhile there is a rich body of evidence, that “forgiveness therapy” may improve psychological health (10–12). A recent meta-analysis found that these interventions reduced anger and hostility, stress and distress, and improved positive affect (12). Similarly, in workplace conflicts, forgiveness was moderately related to less mental health problems and unproductivity (13). The positive effects of forgiveness are partially mediated by reducing stress due to workplace offensiveness, and “forgiveness may be an effective means of coping following being emotionally hurt on the job that may promote good health, well-being, and productivity” (13).

Related research also shows that in terror attack victims the tendency to forgive was associated with problem-focused coping strategies instead of avoidance coping, and problem-focused coping was related to less PTSD symptoms (14). Interestingly, in the same study emotion-focused coping strategies were related to higher PTSD symptoms. For soldiers, active and cognitive processing focused on addressing the underlying problems (which may contribute to their burdening symptoms) might be a more healthy process instead of avoiding feelings. Here we have to assume both, situational demands and conflicts on the one hand, and personal traits (“tendency to forgive”) on the other hand (15).

With respect to self-forgiveness, a recent meta-analysis summarized its effects on physical and mental health and found moderate positive effects on mental health and physical health across 65 and 18 studies, respectively (16). Toussaint et al. (17) reported that in cancer patients and their caregivers self-forgiveness was negatively related with self-blame and distress, but positively with hope. Interestingly, “self-forgiveness was indirectly associated with psychological distress through hope”; an effect that was stronger in the caregiver than in the patients. Even when the caregivers may have the best intentions to care for their patients, they may nevertheless often feel that it was not enough or that they have failed to be more aware, or that they cannot fully help the suffering person. Forgiving oneself can be a complicated process, particularly when one may perceive “guilt” without “fault” (18).

Similar processes can be assumed for soldiers, too, and thus forgiveness as a coping process might be interesting as soldiers have to deal with emotionally burdensome situations and in several cases “moral injury” or inner conflicts. But these processes require an active will to face these problems and find solutions to deal with them. However, it is also true that repressing guilty feelings may prevent the initiation of self-forgiveness, and this could negatively influence recovery and future behaviors in similar situations. Accepting responsibility when things went wrong and also accepting feelings of guilt and shame, even when there was no objective wrong in the concrete situation, is nevertheless painful.

Therefore, it is of importance to clarify whether soldiers do perceive any “needs” to reflect back on their life, to seek release from open aspects of their life, and to talk with others about their fears and worries, and further whether they see any need to forgive others or to forgive oneself. The present study examined if soldiers have unmet reflection, clarification, and forgiveness needs and how strong these needs are. Further the present study clarifies whether these needs are related to stress perception, PTSD symptoms and reduced life satisfaction on the one hand, and religious trust as a resource to cope on the other hand.

## Methods

### Participants

This is a cross-sectional study of German soldiers assessed between November 2011 and February 2012 (*n* = 816 in the first phase) and December 2012 to December 2013 (*n* = 281 in the second phase) (9). Ethical approval was obtained by the IRB of Witten/Herdecke University (#109/2011). The German Ministry of Defense (BMVg, PSZ III 6) approved and registered the study (#2/04/12).

The questionnaires were administered to German soldiers (mainly explosive ordnance disposal unit, military police/personal security and medical services in the first phase, and regular soldiers of army, air force and navy in the second phase) and military personnel treated as in-patients for post-traumatic stress disorder in the German Armed Forces Military Hospital Hamburg (Department of Psychiatry and Psychotherapy). The response rate of the first phase was 38%, while we have no information on the response rate of the second phase.

Data entry was performed at the German Armed Forces Joint Support Command, Cologne, and Bonn.

### Measures

#### Needs to reflect, clarify, and forgive

To measure a person's psychosocial, existential and spiritual needs, we used the SpNQ (19, 20). From this instrument we used five items addressing the needs to (1) reflect back on life (N4W), (2) clarify open aspects (N5W), (3) talk about fears and worries (N2W), (4) forgive someone (N16W), and (5) be forgiven (N17W). These five items can be combined to a single factor (“Forgiveness and Clarification”; explaining 47% of variance) with acceptable internal consistency (Cronbach's alpha = 0.71) in this sample.

From the SpNQ-20 we also took the *Inner Peace Needs* subscale (Cronbach's alpha = 0.73) (20) which uses four items: wish to dwell at places of quietness and peace (N7W), plunge into the beauty of nature (N6W), finding inner peace (N8w), talking with others about fears and worries (N2W). In this sample, the subscale had acceptable internal consistency (Cronbach's alpha = 0.74).

All items were scored with respect to the intensity of needs on a 4-point scale from disagreement to agreement (0—not at all; 1—somewhat; 2—strong; 3—very strong).

#### Life satisfaction

Life satisfaction was measured using the Brief Multidimensional Life Satisfaction Scale (BMLSS) (21). The items of the BMLSS address intrinsic (oneself, life in general), social (friendships, family life), external (work situation, where one lives), and prospective dimensions (financial situation, future prospects) of life satisfaction as a multifaceted construct. The internal consistency of the instrument was found to be good in the validation study (Cronbach's alpha = 0.87) (21). In this study the 10-item version was employed that includes satisfaction with the health situation and abilities to deal with daily life concerns (BMLSS-10). The scale exhibited a good internal consistency in the present sample (Cronbach's alpha = 0.83).

All items were introduced by the phrase “I would describe my level of satisfaction as …,” and scored on a 7-point scale ranging from dissatisfaction to satisfaction (0—terrible; 1—unhappy; 2—mostly dissatisfied; 3—mixed (about equally satisfied and dissatisfied); 4—mostly satisfied; 5—pleased; 6—delighted). The BMLSS-10 sum scores refer to a 100% level (“delighted”). Scores >60% indicate higher life satisfaction, while scores <40% indicate dissatisfaction, and scores between 40 and 60 indicate indifference.

#### Perceived stress scale

Cohen's Perceived Stress Scale (PSS) measures a person's self-perceived stress level in specific situations during the last month (22). Four items of the 10-item version (PSS-10) use a reverse scoring. Internal reliability of the original PSS-10 was moderate (alpha = 0.78) (22). Within this sample, the German language version of the PSS-10 has a good internal reliability (Cronbach's alpha = 0.85).

All items refer to emotions and thoughts and how often one may have felt or thought a certain way. The scores range from 1 (never) to 4 (very often); higher scores would thus indicate greater stress.

#### Stressful military experiences/post-traumatic stress disorders

Stressful military experiences in terms of PTSD were measured with the German version of a modified PTSD Checklist-Military Version (PCL-M) (23). The checklist addresses problems associated with psychological distress that soldiers and veterans may experience such as repeated, disturbing memories, thoughts, images or dreams of a stressful military experience, physical reactions when reminded of a stressful military experience, avoidance of activities or situations because they reminded the soldier of a stressful military experience, being “superalert” or watchful or on guard, etc. (24, 25).

The internal reliability of the 17-item German language version was very good in this sample (Cronbach's alpha = 0.93). For this modified version, the respective items were formulated as whole sentences instead of reduced sentences.

The respective items were scored on a 5-point Likert scale ranging from 1 (not at all) to 5 (extremely). The total symptom severity score may range from 17 to 85. We did not use the checklist to diagnose PTSD, but to screen individuals for perceived stressful experiences.

The PCL-M scores can be categorized in three groups “low” (PCL-M scores 17–33), “moderate” (scores 34–43), and “high” (scores 44–85) (26).

#### Religious trust

To analyze a person's religious Trust, we used a specific subscale of the SpREUK questionnaire (SpREUK is an acronym for the German translation of “Spiritual and Religious Attitudes in Dealing with Illness”) (27–29). This Trust scale has good internal consistency (Cronbach's alpha = 0.84) and addresses a person's trust in spiritual guidance for their life, their feeling of being connected with a higher source, trust in a higher power which carries through whatever may happen, and conviction that death is not an end. In this sample, the scale's internal consistency is good (Cronbach's alpha = 0.88).

The instrument scores items on a 5-point scale from disagreement to agreement [0—does not apply at all; 1—does not truly apply; 2—don't know (neither yes nor no); 3—applies quite a bit; 4—applies very much]. For all analyses, we used the mean scores of the respective scales described above. These scores are based on a scale of 100% (transformed scale score). Scores >60% indicate higher agreement (positive attitude), while scores <40% indicate disagreement (negative attitude), and scores between 40 and 60 an indifferent attitude.

### Statistical analysis

Descriptive statistics as well as analyses of variance, first order correlations and stepwise regression analyses were computed with SPSS 23.0.

Mediation modeling was performed using SPSS 23.0 following the conceptual theory from Hayes (30). The mediation analysis allows the researcher to investigate not only a direct effect from a variable on another but it is also possible to learn the indirect effect that a variable may have in a model. This relationship between variables and their direct and indirect effects on each other is analyzed with mediation models.

Despite the exploratory character of this study, the significance level was set at *p* < 0.001. With respect to the observed correlations (Spearman rho), we regarded *r* > 0.5 as a strong correlation, an *r* between 0.3 and 0.5 as a moderate correlation, an *r* between 0.2 and 0.3 as a weak correlation, and *r* < 0.2 as no or a negligible correlation.

## Results

### Characteristics of enrolled soldiers

The enrolled soldiers (*N* = 1,097) were predominantly male (92%), were living with a partner (76%), and had a Christian affiliation (66%). Most were between 26 and 35 years of age (46%). All further details are depicted in Table 1.

**Table 1:** Characteristics of soldiers in the sample (*N* = 1,097).

**GENDER (%)**	
Men	92
Women	8
**AGE (%)**	
<26 years	12
26–30 years	26
31–35 years	20
36–40 years	14
41–45 years	12
>45years	15
**PARTNER STATUS (%)**	
Living with partner	76
Without partner	23
**RELIGIOUS DENOMINATION (%)**	
Christian	66
Other	1
None	33
**Days at mission (d)**	319 ± 284
**TRAUMATA (%)**	
Psycho-emotional	8
In hospital for treatment	4
**HEALTH ASSOCIATED VARIABLES (MEAN ± SD)**	
Life Satisfaction (BMLSS-10; range: 0–100)	73.5 ± 13.9
Perceived Stress (PSS-10; range: 0–40)	15.4 ± 6.7
PTSD scores (PCL-M; range: 17–85)	27.0 ± 10.7
Religious Trust (SpREUK-10; range: 0–100)	23.0 ± 26.6
Inner Peace Needs (SpNQ; range: 0–3)	1.0 ± 0.8

With respect to quality of life measures, stress perception, and PTSD symptoms scores were low in the sample, while their life satisfaction was in the “satisfied” range (Table 1). Religious Trust was very low, indicating that religiosity had little relevance for most of the soldiers, while their inner peace needs were in the “somewhat” range.

### Needs to reflect, clarify, and forgive

A large percentage (30%) of soldiers had strong to very strong needs to reflect back on their lives, while 51% had no such needs (Table 2). The intention to clarify open aspects of their life (whatever these might be, i.e., perceived guilt, failures, conflicts) was found to be strong/very strong in intensity in 23% of soldiers, while 63% had no such needs. Although the topic was not explicitly addressed, 30% had strong to very strong needs to talk with someone about their fears and worries (56% not), indicating that there are hidden conflicts or inner struggles. These talks are often a first and important step to cope with the underlying burdening situations, experiences, or straining worries.

**Table 2:** Intensity of needs in the sample of soldiers.

	**Intensity of needs**			
	**Not at all (%)**	**Somewhat (%)**	**Strong (%)**	**Very strong (%)**
Reflect back on life (N4W)	51	19	23	7
Clarify open aspects (N5W)	63	14	13	10
Talk about worries and fears (N2W)	56	14	22	8
Forgive someone (N16W)	80	9	7	5
Be forgiven (N17W)	81	7	7	6

Further steps to cope with personal guilt or perceived failures (whether they are true or not) were reflected in the need to be forgiven, or when guilt and failures were attributed to others, intentions to forgive others. However, 12% of soldiers had strong to very strong needs to forgive others (80% not), and 13% strong to very strong need to be forgiven (81% not) (Table 2).

### Needs to reflect, clarify, and forgive and their association with sociodemographic variables

Gender related differences in the intensity of the five needs were found particularly for the need to talk with someone about worries and fears (N2W; *F* = 15.99, *p* < 0.0001) which was stronger in women than in men, and slightly also for the need to forgive someone (N16W; *F* = 7.71, = 0.010) which was lowest in men (Table 3). Regarding age, there were no significant differences in the intensity of needs (data not shown) except that the need to reflect back showed some inconsistent age differences (*F* = 2.53, *p* = 0.019).

**Table 3:** Intensity of needs related to gender and hospital treatment.

		**Reflect back on life (N4W)**	**Clarify open aspects (N5W)**	**Talk with someone about worries and fears (N2W)**	**Forgive someone (N16W)**	**Be forgiven (N17W)**
All soldiers (*n* = 1,084)	Mean	0.87	0.71	0.82	0.37	0.38
	*SD*	1.01	1.04	1.03	0.82	0.86
**GENDER**						
Men (*n* = 999)	Mean	0.87	0.70	0.78	0.35	0.38
	*SD*	1.01	1.03	1.03	0.80	0.85
Women (*n* = 85)	Mean	0.82	0.76	**1.25**	0.59	0.46
	*SD*	0.99	1.11	**1.01**	0.98	0.96
*F*-value		0.16	0.26	15.99	7.71	0.81
*p*-value		n.s.	n.s.	<0.0001	0.010	n.s.
**FAMILY STATUS**						
Married (*n* = 549)	Mean	0.89	0.65	0.81	0.33	0.32
	*SD*	1.02	1.01	1.05	0.76	0.78
With partner (*n* = 273)	Mean	0.70	0.67	0.83	0.30	0.45
	*SD*	0.94	1.02	1.01	0.76	0.95
Divorced (*n* = 52)	Mean	**1.37**	**1.22**	**1.04**	0.71	0.75
	*SD*	**1.01**	**1.22**	**1.03**	1.14	1.15
Single (*n* = 204)	Mean	0.90	0.77	0.75	0.48	0.38
	*SD*	1.01	1.06	1.00	0.91	0.84
*F*-value		7.04	5.09	1.09	5.46	4.57
*p*-value		<0.0001	0.002	n.s.	0.001	0.003
**PHYSICAL TRAUMATA**						
With (*n* = 33)	Mean	**1.06**	0.79	0.97	0.45	0.48
	*SD*	**1.03**	1.08	1.03	0.94	0.94
Without (*n* = 1,054)	Mean	0.86	0.71	0.81	0.36	0.38
	*SD*	1.00	1.04	1.03	0.81	0.86
*F*-value		1.27	0.18	0.72	0.40	0.46
*p*-value		n.s.	n.s.	n.s.	n.s.	n.s.
**MENTAL TRAUMATA**						
With (*n* = 82)	Mean	**1.39**	**1.10**	**1.71**	0.55	0.91
	*SD*	**1.12**	**1.19**	**1.14**	1.03	1.26
Without (*n* = 1,003)	Mean	0.82	0.68	0.75	0.35	0.34
	*SD*	0.98	1.02	0.99	0.80	0.81
*F*-value		24.61	12.26	69.53	4.39	34.46
*p*-value		<0.0001	<0.0001	<0.0001	0.036	<0.0001
**HOSPITAL TREATMENT**						
Hospital (*n* = 41)	Mean	**1.56**	**1.32**	**1.80**	0.73	**1.05**
	*SD*	**1.23**	**1.33**	**1.11**	1.14	**1.32**
At work (*n* = 1,048)	Mean	0.84	0.69	0.78	0.35	0.36
	*SD*	0.99	1.02	1.01	0.80	0.83
*F*-value		20.80	14.71	39.09	8.41	26.00
*p*-value		<0.0001	<0.0001	<0.0001	0.004	<0.0001

*Needs with scores >1.0 were highlighted (bold)*.

Significantly higher needs were found in the rather small group of divorced soldiers, compared to those living with a partner or even those living as singles. However, the needs to forgive or be forgiven have rather low intensity in this group, although these differences are nevertheless statistically significant (Table 3).

Soldiers treated in hospital for trauma had significantly higher needs scores than those at work (“regular duties”), particularly for the needs to talk with others (*F* = 39.1; *p* < 0.0001) and to be forgiven (*F* = 26.0; *p* < 0.0001).

**Figure 1:**
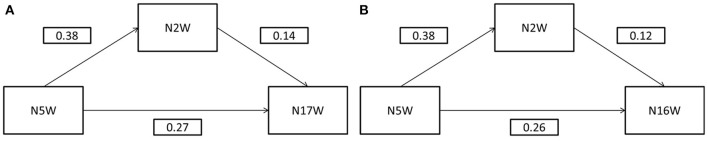
**(A)** Mediation Model for N17W. **(B)** Mediation Model for N16W. N2W, Talk with someone about worries and fears; N5W, clarify open aspects (N5W); N16W, forgive someone; N17W, be forgiven; scores are β values.

To analyze whether talking with someone about worries and fears (N2W) might be a mediator of the effects between soldiers' needs to clarify open aspects (N5W) and to be forgiven (N17W) or to forgive others (N16W), we performed a simple regression to N17W and N16W. Both models and coefficients are presented in Table 4. In both models, all three statistical paths were significant—total, direct and indirect effects. The indirect effects of N5W on N17W mediated by N2W is β = 0.05 (*p* < 0.001) and of N5W on N16W mediated by N2W is β = 0.04 (*p* < 0.001)—they are small yet relevant and represent around 18 and 15%, respectively of the direct effect model's coefficients (Figures 1A,B).

**Table 4:** Model coefficients for N2W as a mediator.

**Variables**	**M** = **Mediator (N2W)**			**Y (N17W)**			**Y (N16W)**		
	**β**	**SE**	***p*-value**	**β**	**SE**	***p*-value**	**β**	**SE**	***p*-value**
X (N5W)	0.38	0.03	<0.001	0.27	0.02	<0.001	0.26	0.02	<0.001
Mediator (N2W)	–	–	–	0.14	0.02	<0.001	0.12	0.02	<0.001
Constant	0.55	0.03	<0.001	0.08	0.03	0.01	0.09	0.03	<0.01
	*R*^2^ = 0.14			*R*^2^ = 0.17			*R*^2^ = 0.16		
	*F_(1, 1, 075)_* = 179.42			*F_(2, 1, 074)_* = 113.05			*F_(2, 1, 072)_* = 105.05		
	*p* < 0.001			*p* < 0.001			*p* < 0.001		

### Interconnections between needs to reflect, clarify, and forgive and health indicators

One may assume that soldiers who have experienced burdening situations or interpersonal conflicts may have stronger needs to reflect, clarify, and forgive than soldiers who do not have experience with such trauma or who are able to cope. In fact soldiers who are treated in the Department of Psychiatry and Psychotherapy had significantly higher needs to reflect, clarify, talk, forgive, and be forgiven (Table 3). Here, the strongest differences were found for the needs to talk about their fears and worries, to be forgiven, and to reflect back on their life, while the difference to forgive someone were less strong compared to the other soldiers. This may indicate that these soldiers have to deal with inner conflicts (“fears and worries”), may perceive failures and guilt (“be forgiven”) and still have to deal with burdening experiences (“reflection” and “clarification”).

Correlation analyses (Table 5) indicate that the needs to clarify open aspects are moderately related to the needs to talk with someone about fears and worries, to be forgiven and to forgive on the one hand, and moderately related with reduced life satisfaction, and further weakly related with stress perception and PTSD symptoms on the other hand. The intended clarification process is clearly related with the resolving talks and own forgiveness (whatever the underlying reason might be). In contrast, the need to reflect back on life is only weakly related to both forgiveness needs, and weakly to low life satisfaction, stress perception and PTSD symptoms.

**Table 5:** Correlation between needs and indicators of health and spirituality.

	**Reflect back on life (N4W)**	**Clarify open aspects (N5W)**	**Talk with someone about worries and fears (N2W)**	**Forgive someone (N16W)**	**Be forgiven (N17W)**	**Forgiveness/Clarification Needs Scale**
Reflect back on life (N4W)	1.000				
Clarify open aspects (N5W)	0.298^**^	1.000			
Talk about worries and fears (N2W)	0.309^**^	0.358^**^	1.000		
Forgive someone (N16W)	0.216^**^	0.357^**^	0.253^**^	1.000	
Be forgiven (N17W)	0.238^**^	0.359^**^	0.259^**^	0.466^**^	1.000
Inner Peace Needs (SpNQ)	0.394^**^	0.366^**^	0.647^**^	0.298^**^	0.289^**^	0.635^**^
Stress perception (PSS)	0.239^**^	0.289^**^	0.348^**^	0.216^**^	0.250^**^	0.413^**^
PTSD symptoms (PCL-M)	0.259^**^	0.251^**^	0.322^**^	0.152^**^	0.209^**^	0.373^**^
Religious Trust (SpREUK)	0.149^**^	0.143^**^	0.194^**^	0.125^**^	0.129^**^	0.231^**^
Life satisfaction (BMLSS)	−0.257^**^	−0.300^**^	−0.328^**^	−0.152^**^	−0.216^**^	−0.393^**^
Family life	−0.178^**^	−0.229^**^	−0.217^**^	−0.136^**^	−0.147^**^	−0.281^**^
Friends	−0.134^**^	−0.143^**^	−0.175^**^	−0.031	−0.078^**^	−0.192^**^
Work place	−0.105^**^	−0.085^**^	−0.142^**^	−0.067	−0.094^**^	−0.148^**^
Myself	−0.240^**^	−0.239^**^	−0.270^**^	−0.134^**^	−0.219^**^	−0.345^**^
Where I live	−0.117^**^	−0.223^**^	−0.177^**^	−0.059	−0.164^**^	−0.226^**^
Life in general	−0.221^**^	−0.265^**^	−0.275^**^	−0.142^**^	−0.198^**^	−0.332^**^
Financial situation	−0.121^**^	−0.206^**^	−0.136^**^	−0.124^**^	−0.150^**^	−0.223^**^
Future perspectives	−0.194^**^	−0.179^**^	−0.199^**^	−0.102^**^	−0.146^**^	−0.254^**^
Health situation	−0.101^**^	−0.134^**^	−0.200^**^	−0.075	−0.097^**^	−0.193^**^
Management daily life concerns	−0.179^**^	−0.255^**^	−0.296^**^	−0.160^**^	−0.194^**^	−0.326^**^

** *p < 0.0001 (Spearman rho); moderate to strong correlations were highlighted (bold)*.

### Needs to reflect, clarify, and forgive and their association with health indicators

The need to talk with someone about one's own fears and worries is moderately related to stress perception, PTSD symptoms and low life satisfaction, while these talks may not necessarily relate to needs to forgive or be forgiven, as these associations were weak. Rather it is the intention to clarify “open aspects in life” which is much more related to forgiveness. Interestingly, both forgiveness needs are marginally to weakly related to stress perception, PTSD symptoms or reduced life satisfaction (Table 5).

The intentions to reflect and clarify are moderately related to soldiers' needs for inner peace, which is sound from a theoretical point of view as it indicates strategies to resolve problems and struggles, to let go and to find states of inner peace again. Also both forgiveness needs are weakly to moderately related to inner peace needs, but much weaker.

Detailed analyses with the sub-dimensions of life satisfaction revealed several weak associations, particularly with satisfaction with oneself and life in general, and abilities to manage daily life concerns (Table 5). These were mainly related with the clarification and talking needs.

Religious trust, as an indicator of intrinsic religiosity, was marginally related to the needs to reflect, clarify and forgive (Table 5), and is thus not of outstanding relevance as a resource to cope.

The forgiveness/clarification needs scale was strongly related with inner peace needs, and moderately with stress perception, PTSD symptoms and reduced life satisfaction (particularly with satisfaction with oneself, life in general, and abilities to manage daily life concerns), and weakly with religious trust (Table 5).

### Predictors of needs to reflect, clarify, and forgive

Because both stress related variables (PSS and PCL-M), but also life satisfaction (with the three more relevant sub-dimensions), religious trust, and also being divorced were significantly related to the five needs variables, we performed stepwise regression analyses to identify the best predictors (Table 6).

**Table 6:** Predictors of needs to reflect, clarify, and forgive.

	**β**	**T**	***p***
**Dependent variable: N4W**			
***F* = 29.6, *p* <0.0001; *R*^2^ = 0.13**			
(Constant)		3.077	0.002
PTSD symptoms (PCL-M)	0.165	4.513	<0.0001
Satisfaction with myself (BMLSS)	−0.135	−3.816	<0.0001
Religious Trust (SpREUK)	0.108	3.611	<0.0001
Divorced	0.099	3.330	0.001
Stress symptoms (PSS)	0.090	2.303	0.021
**Dependent variable: N5W**			
***F* = 39.9, *p* < 0.0001; *R*^2^ = 0.16**			
(Constant)		3.379	0.001
Life satisfaction (BMLSS)	−0.185	−4.734	<0.0001
Stress symptoms (PSS)	0.138	3.471	0.001
Religious Trust (SpREUK)	0.102	3.458	0.001
PTSD symptoms (PCL-M)	0.107	2.871	0.004
Divorced	0.079	2.694	0.007
**Dependent variable: N2W**			
***F* = 60.8, *p* < 0.0001; *R*^2^ = 0.24**			
(Constant)		2.333	0.020
PTSD symptoms (PCL-M)	0.142	3.628	<0.0001
Stress symptoms (PSS)	0.174	4.626	<0.0001
Religious Trust (SpREUK)	0.167	5.920	<0.0001
Life satisfaction (BMLSS)	−0.150	−4.064	<0.0001
Mental traumata	0.115	3.603	<0.0001
**Dependent variable: N16W**			
***F* = 31.6, *p* < 0.0001; *R*^2^ = 0.09**			
(Constant)		−3.142	0.002
Stress symptoms (PSS)	0.249	8.168	<0.0001
Religious Trust (SpREUK)	0.130	4.270	<0.0001
Divorced	0.065	2.144	0.032
**Dependent variable: N17W**			
***F* = 32.9, *p* < 0.0001; *R*^2^ = 0.14**			
(Constant)		−0.080	0.936
PTSD symptoms (PCL-M)	0.181	4.962	<0.0001
Stress symptoms (PSS)	0.145	3.779	<0.0001
Religious Trust (SpREUK)	0.097	3.275	0.001
Satisfaction with Life in general (BMLSS)	−0.095	−2.714	0.007
Divorced	0.076	2.558	0.011
**Dependent variable: FCNS**			
***F* = 89.1, *p* < 0.0001; *R*^2^ = 0.31**			
(Constant)		2.906	0.004
Stress symptoms (PSS)	0.209	5.834	<0.0001
PTSD symptoms (PCL-M)	0.203	6.053	<0.0001
Religious Trust (SpREUK)	0.179	6.732	<0.0001
Life satisfaction (BMLSS)	−0.181	−5.149	<0.0001
Divorced	0.099	3.738	<0.0001

*FCNS, Forgiveness/Clarification Needs Scale. In none of the models: Satisfaction with management of daily life concerns was significant*.

Soldiers' needs to reflect back on life (N4W) were predicted best by PTSD which explains 8% of variance; reduced satisfaction with oneself, religious trust, stress symptoms and being divorced would add further 5% of explained variance.

Needs to clarify open aspects (N5W) were predicted best by reduced life satisfaction which explains 11% of variance; stress perception, religious trust, PTSD symptoms and being divorced would add 5% of further explained variance.

Needs to talk with someone about worries and fears (N2W) were predicted best by PTSD symptoms, which explains 15% of variance; further 4% were explained by stress perception, and additional 4% by religious trust, low life satisfaction and mental trauma.

Soldiers' needs to forgive someone (N16W) were predicted best by stress perception, which would explain 7% of variance, while religious trust, PTSD symptoms and being divorced would add further 2% of explained variance. This prediction model is much too weak to draw any relevant conclusions.

Needs to be forgiven (N17W) was predicted best by PTSD symptoms, which would explain 10% of variance, while stress perception, religious trust, low satisfaction with life in general, and being divorced would add further 4% of explained variance.

With respect to the condensed forgiveness/clarification needs scale, the best predictors were stress perception, which would explain 20% of variance, PTSD symptoms would further explain 5% of variance, and reduced life satisfaction, religious trust and being divorced would add further 6% of explained variance.

### Stress perception and PTSD symptoms and intensity of needs

When soldiers stress perception and PTSD symptoms were identified as relevant variables associated with the needs to reflect, clarify, and forgive, it is worthwhile to clarify their role in this process.

Both stress related variables are moderately associated (*r* = 0.47). Soldiers with high stress level may have high PTSD symptoms, but it does not have to be that way. In fact, in this sample 37% of soldiers with high stress scores had high PTSD symptoms, 26% moderate PTSD symptoms and 38% low PTSD symptoms. Those with moderate stress scores have moderate or high PTSD scores of 13 and 5%, respectively. Thus, both variables might be related, but may refer to different situations and underling processes.

As shown in Table 7, soldiers with high stress perception scores had significantly higher needs to talk about fears and worries, reflect back on their life and to clarify open aspects; also their forgiveness needs are higher compared to those with moderate or low stress scores, but the intensity is nevertheless rather low. The pattern for soldiers with PTSD symptoms is similar (Table 7), but here also persons with moderate PTSD scores had relatively high needs to reflect and talk with someone about their fears and worries, while their forgiveness needs are similar to that of soldiers with PTSD symptoms.

**Table 7:** Needs related to stress perception and PTSD symptoms.

		**Reflect back on life (N4W)**	**Clarify open aspects (N5W)**	**Talk with someone about worries and fears (N2W)**	**Forgive someone (N16W)**	**Be forgiven (N17W)**	**Forgiveness /Clarification Needs Scale**
All soldiers (*n* = 1,089)	Mean	0.87	0.71	0.82	0.37	0.38	0.63
	*SD*	1.01	1.04	1.03	0.82	0.86	0.65
**STRESS PERCEPTION (PSS)**							
Low (score 0–9) (15%)	Mean	0.58	0.37	0.45	0.12	0.17	0.34
	*SD*	0.87	0.81	0.84	0.44	0.59	0.49
Moderate (score 9–22) (70%)	Mean	0.81	0.64	0.72	0.35	0.32	0.57
	*SD*	0.96	0.98	0.96	0.78	0.76	0.57
High (score 23–37) (15%)	Mean	**1.43**	**1.39**	**1.66**	0.73	0.90	**1.22**
	*SD*	**1.13**	**1.24**	**1.12**	1.12	1.27	**0.65**
*F*-value		35.3	49.1	75.3	23.9	38.4	101.5
*p*-value		<0.0001	<0.0001	<0.0001	<0.0001	<0.0001	<0.0001
**PTSD SYMPTOMS (PCL-M)**							
Low (score 2–32) (75%)	Mean	0.75	0.62	0.64	0.31	0.28	0.52
	*SD*	0.94	0.98	0.92	0.74	0.72	0.57
Moderate (score 33–42) (13%)	Mean	**1.06**	0.79	**1.21**	0.41	0.50	0.80
	*SD*	**1.02**	1.07	**1.09**	0.85	0.92	0.64
High (score 43–79) (9%)	Mean	**1.51**	**1.51**	**1.78**	0.86	**1.22**	**1.37**
	*SD*	**1.21**	**1.28**	**1.20**	1.27	**1.38**	**0.88**
*F*-value		28.0	31.9	70.4	19.1	54.0	87.2
*p*-value		<0.0001	<0.0001	<0.0001	<0.0001	<0.0001	<0.0001

*Needs with scores >1.0 were highlighted (bold)*.

## Discussion

While it is true that most soldiers avoid talking about burdening experiences and try to find private ways to silently cope to avoid stigmatization, it is important to find *indicators* to identify persons in need (31). Addressing and supporting soldiers' psychosocial, existential and spiritual needs might help soldiers who are in need of assistance (9). In the present study we assumed that some persons may need to reflect back on life with the intent to clarify past conflicts or burdening situations particularly when these needs are still vital and have a negative impact on life concerns. As a consequence, these soldiers may have the need to talk with others about their fears and worries, and needs to be forgiven or to forgive others. Therefore, it is important to assess who may have these needs, which variables may contribute to these needs, and how these needs are related to quality of life outcomes.

We found that among German soldiers about one third have strong reflection, clarification and talking needs, while the more explicit forgiveness needs were expressed by only 13% with strong emphasis and 80% do not have forgiveness needs at all. This is consistent with research showing moderate levels of a personality trait that describes a characterological tendency to take offense at others' behaviors (32), but it contrasts considerably with research showing relatively high levels of forgiveness needs in pain patients (33). Interestingly, the effect of the clarification needs on soldiers' forgiveness needs was mediated to some extent by their need to talk with someone about worries and fears. Talking about their problems seems to be crucial. This may reflect an underlying need for social support that may explain the association between clarification needs and forgiveness needs. That is, it is in seeking clarification that soldiers invoke social support resources that in turn facilitate forgiveness needs. Indeed, forgiveness has been theorized to be closely connected to social support because forgiveness may be a crucial component in maintaining social ties (34). These aforementioned “starter needs” (reflection, clarification, and talking) were particularly high in divorced soldiers and soldiers with mental trauma. The need to talk about fears and worries was strongly related to inner peace needs and moderately with stress perception and PTSD symptoms. The strongest talking needs were found in soldiers with mental trauma and in those who were treated for mental traumata in the hospital, and the best predictors were stress, PTSD symptoms, mental trauma, reduced life satisfaction, and religious trust. This means that these indicators point to the fact that they have experienced difficult and burdening situations or conflicts which are still challenging for their mental stability. Even when they may have started to talk with others about their fears and worries and tried to find strategies to cope, this specific need is still unmet and they require further support.

Only a small percentage of soldiers had needs to be forgiven which might imply that they were coping with failure, guilt or shame with other methods. But it is important to note that soldiers may be coping in maladaptive ways with these self-condemning feelings (35) and the need to be forgiven could motivate a process of self-forgiveness or seeking forgiveness through a religious ritual or finding other ways to feel forgiven by others for wrongdoing. Often these forgiveness motives go hand in hand (36). Similarly, a small percentage of soldiers had needs to forgive others which might imply that others may have failed in specific situations or are the cause of conflicts but soldiers were effectively dealing with these interpersonal issues. But again, not all methods of coping with conflicts and interpersonal issues are adaptive. Revenge-seeking, condoning, denial, etc. may all appear on the surface as effective means of dealing with problems caused by other people, but often these are maladaptive or perpetuate a cycle of harm (37). For 80% of soldiers these needs are not perceived, while for 12–13% these needs are strong to very strong, and for 7–9% somewhat relevant. Although persons with mental trauma or a hospital stay had significantly higher needs to be forgiven, the intensity of this need was rather moderate compared to the “no-needs” scores of the other soldiers. Predictor analyses would indicate that PTSD symptoms and stress perception are of some relevance as they explained at least 12% of variance. However, for soldiers' needs to forgive others, the prediction model would point to stress perception as relevant variable, but the predictive power is much too weak to have much confidence in. Nevertheless, this is consistent with research showing connections between changes in stress and changes in forgiveness (38).

The present simple instrument could be helpful in identifying soldiers in need. The five analyzed indicator items could be used as a single scale termed “Forgiveness/Clarification Needs.” This condensed scale had acceptable internal consistency (Cronbach's alpha = 0.74), and stress and PTSD symptoms were the best predictors of these needs (explaining 25% of variance). Reduced life satisfaction, religious trust and being divorced added an additional six percent of explained variance.

Why religious trust was among the weaker predictors is unclear. Thirty-three percent of soldiers in this sample had no religious affiliation and 66% were nominally Christians. Nevertheless, their religious trust scores were rather low indicating that this resource is not of general relevance, but may be important for only some soldiers. In fact, 13% had scores >60 indicating religious trust. Even in this small group, forgiveness needs were weak (N16W score: *M* = 0.55, *SD* = 0.93; N17W: *M* = 0.52, *SD* = 0.93), but significantly higher compared to other soldiers (*F* = 7.0, *p* = 0.001 and *F* = 3.9, *p* = 0.020, respectively), while their needs to talk with others about fears and worries were much stronger (N2W: *M* = 1.20, *SD* = 1.15), and significantly higher compared to the others (*F* = 12.0; *p* < 0.0001). All these needs were only marginally related to religious trust (*r* < 0.20). For religious persons one could expect that the need to be forgiven is a religious matter in terms of confession and repentance, but it seems not in this sample of relatively young soldiers. This finding again underscores what is seemingly a paradox in the relationship between religion and forgiveness (39). In a sense, religious people ought to value forgiveness more highly, but this does not always translate into greater levels of forgiveness of specific people or events and in the present case religious affiliation may not translate into greater perceived needs to forgive others. Thus, religious trust as a resource may have some marginal influence, but its relevance for the reflection and clarification processes should not be over-emphasized.

It seems that reflecting back on life and talking about fears and worries is a strategy to cope with burdening experiences, but not necessarily related to the perception of one's *own* guilt, failures, or moral injury which may subsequently result in needs to be forgiven. Instead it seems that the *intensity* of burdening experiences (PTSD symptoms) is related with needs to let go feelings of guilt, perceived failures, or moral injury as it is clearly related to the needs to be forgiven rather than to forgive others (Table 7). One may assume that soldiers' Inner Peace needs might motivate actual forgiveness—but not necessarily *needs* to forgive. This could be seen as a pathway to health which should be addressed in future studies.

### Limitations

We have no specific information about the underlying causes of soldiers' burdening situations or conflicts resulting in the expression of these needs. Whether these needs may have arisen from moral injury, personal failures, or other reasons of perceived guilt, weakness, or shame remains unclear and was not focus of this study. This remains to be addressed in future studies. We had only limited access to soldiers treated for PTSD, and thus a specific study among this group of soldiers would be of importance. Further, this is a cross-sectional study and inferences about causality cannot be made. Needs may precede or follow the development of burdening situations and conflicts. As this is a sample of German soldiers, generalizations to broader populations of civilians should not be made.

## Conclusions

The process of life reflection and subsequent intention to solve conflicting situations and experiences can be considered as a process to cope with one's own failures, guilt, and mistakes. It should be noted that these needs, which were of strong relevance for up to one-third of soldiers, were significantly stronger in soldiers with trauma. Addressing unmet needs may help them to communicate and to reject the stigma of weakness.

## Author contributions

AB has designed the study, performed statistical analyses and has written the manuscript. DR performed mediator analyses. LT contributed to write the manuscript.

### Conflict of interest statement

AB and DR are employees of the Witten/Herdecke University and were never employed by any military organization. In 2011, AB was appointed as a Technical Team Member of the NATO panel HFM-195 on “Integrative Medicine Interventions for Military Personnel.” The authors disclose any competing interests. The manuscript was approved by the German Ministry of Defense (Reg-Nr. 2/04/12; PIII5 20 July 2018). The remaining author declares that the research was conducted in the absence of any commercial or financial relationships that could be construed as a potential conflict of interest.
